# Role of Edaravone as a Treatment Option for Patients with Amyotrophic Lateral Sclerosis

**DOI:** 10.3390/ph14010029

**Published:** 2020-12-31

**Authors:** HaEun Cho, Surabhi Shukla

**Affiliations:** Department of Pharmaceutical Sciences, College of Pharmacy, Larkin University, 18301 N Miami Ave, Miami, FL 33169, USA; HCho@myularkin.org

**Keywords:** edaravone, ALS, neuroprotection, Rizuole, neurophathology, ROS

## Abstract

Amyotrophic Lateral Sclerosis (ALS), also known as Lou Gehrig’s disease, is a progressive and fatal neurodegenerative disease that leads to a loss of muscle control due to nerve cells being affected in the brain and spinal cord. Some of the common clinical presentations of ALS include weakness of muscles, changes in behavior, dysfunction in speech, and cognitive difficulties. The cause of ALS is uncertain, but through several studies, it is known that mutations in SOD1 or C9orf72 genes could play a role as a factor of ALS. In addition, studies indicate that an excessive amount of free radicals, the reactive oxygen species (ROS), leads to neuronal damage by the peroxidation of unsaturated fatty acids in the neuronal cells. Edaravone, the newly approved antioxidant drug for ALS, halts the progression of ALS in the early stages through its cytoprotective effect and protects the nerves by reducing ROS. In this review, different aspects of ALS will be discussed, including its pathology, genetic aspect, and diagnosis. This review also focuses on edaravone as a treatment option for ALS, its mechanism of action, and its pharmacological properties. Clinical trials and adverse effects of edaravone and care for ALS patient are also discussed.

## 1. Introduction

Amyotrophic Lateral Sclerosis was first diagnosed in 1824 in a patient named Charles Bell, but it became more widely known after the death of a baseball player, Lou Gehrig, hence the name “Lou Gehrig’s disease” [1,2]. In the past, Amyotrophic Lateral Sclerosis (ALS) was known purely as a motor neuron degeneration that affects the brain and spinal cord and eventually leads to paralysis and possibly death from respiratory failure and complications from immobility. However, it is now defined as a multisystem neurodegenerative disorder with the disease characterized at different levels: clinical, neuropathological, and genetics [3].

In ALS patients, as the brain and spinal cord lose the ability of the muscle control due to both the upper and lower motor degeneration, they result in some common clinical symptoms of weakness of muscles that usually begins in the extremities such as limb muscles, more commonly in distal muscles [2]. Patients may also have psychological and cognitive difficulties. They may exhibit depression, maladaptive social behavior, and involuntary emotions, as well as swallowing difficulties. Depending on each of the patients, many differences were seen in the disease onset, age onset, and the rate of disease progression [4]. These symptoms could also categorize ALS into two different types based on the clinical feature or the region at the onset: limb-onset and bulbar-onset. Limb-onset refers to symptoms starting in the limbs, in the arms or legs, whereas bulbar-onset is when the patient begins having a speech or swallowing difficulty [5].

The assembled evidence display how oxidative stress is involved in a number of diseases, including ALS, Parkinson’s disease, Alzheimer’s disease, and many other neurodegenerative diseases. Reactive oxygen species (ROS) are produced by cellular enzymes that damage components of the cells and increase inflammation. An increase of ROS is also linked to low-density lipoprotein (LDL) oxidation, thus leading to the abnormal functioning of endothelial cells in cardiovascular diseases [6].

As the oxidative stress and increase in ROS are involved in various pathways of human diseases, new perception and comprehension of these linked processes will pave the way in developing new therapies and treatments for neurodegenerative diseases [6].

Since ALS is a progressive disease, it is known that most of the patients with ALS die within 2–5 years after their diagnosis of the disease. However, in the past, riluzole was approved to treat ALS for delaying the onset of the disease and increasing the survival time by several months. Recently, in 2017, edaravone was approved by the FDA to be the second drug for treating ALS [7]. Edaravone, known as a free radical scavenger, reduces oxidative stress, playing a role in the disease progression and having a cytoprotective effect on the nerve cells to protect the degeneration of neuronal cells [8].

## 2. Epidemiology of ALS

There are two types of Amyotrophic Lateral Sclerosis: ALS familial (fALS) and sporadic ALS (sALS). Clinically, it is difficult to distinguish between the two, but most of the ALS cases are known to be sALS [9]. No other exact defined etiology was found for the rest of the cases until 2014. However, it was later found that there are other factors involved that contribute to the diagnosis of ALS such as environmental factors and aging-related dysfunction [7]. Recent studies from Europe showed the incidence of ALS as 0.6–3.8 per 100,000 persons and prevalence between 4.1 and 8.4 per 10,000 persons. It was estimated to be higher than a prevalence of 10–12 per 100,000 in Europe, with an incidence of 1.75–3 per 100,000 persons per year [3]. The prevalence of ALS varies between countries. However, over time, it was seen that there is an increase in the number of patients who are getting diagnosed with ALS [10].

The National ALS Registry in the United States gathered information regarding the prevalence of ALS through self-reporting on a web portal that identified 16,583 persons with ALS across different administrative data: Medicare, Veterans Health Administration, and Veterans Benefits Administration. The 2015 estimated prevalence of ALS cases was 5.2 per 100,000 population. The lowest prevalence of ALS cases was in the age range of 18–39 years with 0.5 per 100,000 persons, and the highest was in the age range of 70–79 years with 20.2 per 100,000 persons. There was also a higher prevalence in male compared to female, with the ratio of 1.6:1, and there was a higher prevalence in whites with 5.4 per 100,000 persons compared to that in blacks with 2.3 per 100,000 persons (Table 1) [7].

## 3. Clinical Pathology of ALS

**Figure 1:**
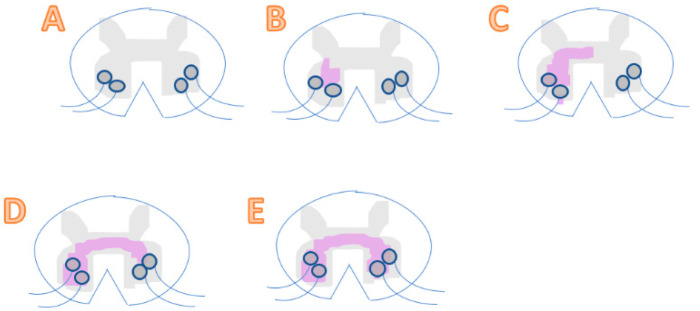
(**A**) A healthy spinal cord with neurons unharmed. (**B**) A pre-symptomatic stage where there is a presence of a local motor neuron dysfunction in the spinal cord, without any clinical symptoms yet. (**C**) Represents a spinal cord during its early symptomatic stage. Spreading of dysfunction in motor neurons occurs and starts showing symptoms. (**D**) A mid-symptomatic stage where dysfunction and degeneration propagate throughout adjacent neurons and regions, thus showing regional dysfunction of neurons. (**E**) The final stage where toxicity transmits to another region and results in a systemic dysfunction of motor neurons. At this late stage, the patient is diagnosed with severe ALS.

ALS involves its neuropathological hallmark of dysfunction of the lower motor neurons in the brain and spinal cord, leading to a loss of motor functions due to oxidative stress. More specifically, uncontrolled oxidative stress causes damage in lipids and DNA. In contrast, ROS is needed to produce advantageous biological effects. Therefore, the correct balance and maintaining homeostasis of free radicals is required for our health [6]. Below is a clinical observation of ALS progression showing random initiation and spreading to nearby regions (Figure 1) [11].

There are two levels of ALS that can be observed: contiguous spread and network spread. Contiguous spread is when it is spreading from side-to-side, being independent of the synaptic connection. Network spread is when connected networks spread from end-to-end, being dependent of synaptic connection. Observing the spread of the motor neuron degeneration is difficult, as it can only be seen clearly through autopsy, as well as being able to see a loss of motor neurons and the atrophy [9].

An increase in the concentration of 3-nitrotyrosine (3-NT), which is a specific marker for oxidative stress that is related to the neuronal degeneration, could also be seen [12]. This is one of the reasons why it is very demanding to determine when the patient specifically started having ALS and see the changes throughout the time period of the disease. However, the level of the progression can be approximately measured by seeing the degree of involvement of the Upper Motor Neuron (UMN) and Lower Motor Neuron (LMN), the regions of the body that has been affected, the degree of involvement or degeneration of other body regions, and the progression rate of the degeneration [13].

## 4. Genetics and Neuropathology of ALS

ALS also possesses a neuropathological hallmark: motor neuron degeneration and loss of axons in the spinal cord. Even though the method of initiation of the neuronal degeneration and the progression toward apoptosis is unclear, Martin hypothesized the stages of apoptosis of motor neurons in ALS. The first stage, chromatolysis, happens where the Nissl bodies dissolute in the cell [2]. Then, in the second stage, somatodendritic attrition happens, and DNA double-strand breaks accumulate [12]. Lastly, apoptosis occurs, where the motor neuron finally dies and loses its function [2].

There are several other diseases, such as Frontotemporal Dementia (FTD) and Alzheimer’s disease (AD), that share common neuropathological features with ALS: the presence of atrophy of neurons in brains [2]. Looking at the relationship between ALS and FTD, it was found that the diagnosis of FTD was made in 10–15% of the cases in ALS patients, proving how they share similar pathology and overlapping clinical signs. Frontotemporal dementia also presents behavioral changes and impairment in language, which could also be presented in ALS patients [3]. Genetic factors are also shared between the two diseases, showing approximately 15% of ALS patients having a family history of either ALS or FTD with a specific variant of superoxide dismutase 1 (SOD1) taking part in up to 13–20% of familial ALS. The journal written by Hardiman and Van Den Berg addresses the potential benefit of reducing the expression of SOD1 in patients who have the SOD1 mutation and ALS with the administration of tofersen, which is an antisense oligonucleotide [14]. A study carried out by Miller et al. 2020 showed two patients receiving a single intrathecal dose of microRNA that targets SOD1; clinically, there seemed to be no huge difference in the level of SOD1 between the two patients. However, a post-mortem study was carried out to measure the SOD1 level, showing a reduction of SOD1 in one of the patients. These studies demonstrated the possibility of treating ALS with the viral-mediated intrathecal insertion of microRNA in patients with the mutation of SOD1, but further study would need to be done to see a clear, definite efficacy of the treatment. Furthermore, other studies and trials are ongoing regarding the treatments with other genetic forms of ALS other than SOD1 [15].

## 5. Diagnosis of ALS

ALS cannot be diagnosed with a specific diagnostic test, but signs of UMN and LMN can be used to identify ALS with the recognition of disease progression throughout different parts of the body. Some LMN signs and symptoms involve weakness and atrophy, with UMN signs and symptoms involving spasticity and hyperreflexia [16]. However, before reaching a definitive diagnosis, there may be some hindrance due to not being able to have a clear onset of symptoms, with the disease itself being progressive, as well as due to uncommon symptoms that are seen. In the end, a delay in the diagnosis hinders patients from having the appropriate treatment and therapies at the right time. As a result, it is important for the patient to have a routine checkup. To name a few, there are nerve conduction studies, such as sensory nerve conduction studies that exclude other degenerative from the ALS, and an electromyography that identifies the loss of LMN [17].

There are specific criteria for the diagnosis of ALS known as the El Escorial criteria [18]. According to El Escorial criteria, the diagnosis of ALS relies on the existence of the following:

1. Clinical evidence of deterioration of lower motor neurons (in spinal cord and brainstem).
2. Clinical evidence of deterioration of upper motor neurons (in brain).
3. Continuous spread of symptoms within a region to other regions with no sign of other disease processes by electrophysiological, pathological, and neuroimaging evidences.

ALS severity also involves using The Revised ALS Functional Rating Scale (ALSFRS-R), which is a system or an assessment that measures the degree of ALS patients’ functional impairment. Twelve items are included for the evaluation—assessing bulbar, motor and respiratory function—which are scored from 0 to 4 each, with 0 referring to not functional at all and 4 referring to fully functional [19].

## 6. Risk Factors

There is research being performed to assess the risk factors for ALS. Age, male sex, and genetics or a past family history of ALS have been known as risk factors. The risk factors could also be divided into genetic factors and environmental factors. Regarding the genetic factor, SOD1 mutations are the first contributing factor to the growth of ALS. SOD1, a cytosolic enzyme, is able to eliminate excess free radicals and convert them to O₂ and H₂O₂. However, when there is a mutation of the SOD1 gene, it builds up and clusters in mitochondria, resulting in dysfunction and finally, death of the neurons [20]. Increasing evidence indicates that many environmental factors such as exposure to pesticides, heavy metals, and herbicides can be a risk factor for developing ALS. The exposure of heavy metals such as lead, cadmium, iron, mercury, and selenium has been studied widely as an environmental risk factor for ALS. Heavy metals deplete thiol containing antioxidants and enzymes in the cells, thereby increasing the oxidative stress in the cells. Previous studies showing lead exposure measurement in patients with ALS suggest lead to be one of the main environmental factors contributing to development of ALS [21,22]. A regional United States case-control study to assess the environmental risk factors for ALS shows that head trauma, electromagnetic fields, and certain occupations such as holding a job in mechanics, construction, and painting can also increase risk factors for developing ALS [23].

## 7. Patient Management and Care

Even though there are different treatment options for patients diagnosed with ALS, they are mostly for symptom improvement and increasing quality of life. As ALS remains incurable, they focus on the patients’ quality of life. Patients managed in a specialized clinic compared to those managed in a general clinic had a better outcome and better quality of life. This could have been due to having more access to effective resources and more focused care of the patients [17]. As respiratory function and nutrition are the main cause of death in ALS patients, they remain crucial concerns for patients, so the patients are recommended to pay special attention to respiratory management and palliative care in general. Non-invasive ventilation betters the patient’s quality of life, and taking sufficient caloric and fluid intake also improves quality of life. Malnutrition plays a role as a key contributing factor of ALS that is caused by numerous other factors. It can be solved by the insertion of a gastrostomy tube that allows enough food and fluid intake for the patient, thus inhibiting further weight loss. Other than dysfunction in respiratory and malnutrition, there could be many other symptoms that patients may suffer from, and it is crucial to relieve those symptoms for alleviating pain and bettering patients’ quality of life [17].

Not only improving the quality of life of a patient is important, but also counseling on end-of-life is a crucial element for both the patients and their families. According to the American Academy of Neurology quality measure, having a “good death” is what is best for the patients, and it is considered a major factor of patient care. The early stage in the progression of the disease is the best time to hold the conversation with the patients regarding their end-of-life decisions. That way, the patients and their family members can agree and make decisions earlier on rather than facing the problem later on and then having to delay the whole process of patient care. In other words, emergency situations and undesirable scenarios can be avoided just by having a conversation about the end-of-life of the patient: differentiating between the wanted and the unwanted process [16].

Making early decisions and being aware of patients’ preferences not only helps the patients and their family members, but it also aids the physicians in their decision-making regarding the services and care for the patients. For example, the patient may be interested in palliative and hospice care rather than taking medications and prolonging the treatment for a lifetime. Discussions regarding the patient’s quality of life should be encouraged, and having conversations about the end-of-life decision making early will be the best for the family members and the physicians but most importantly for the patients themselves [16].

## 8. Treatment

Symptomatic treatments and patient management are the essential foundation of management in ALS patients, as treating the symptoms may also affect the survival rate of patients and the overall quality of life of patients. For example, respiratory function and nutrition could be the main concern; therefore, it is crucial to treat these symptoms first [8]. Another example could be using muscle relaxant and speech therapy to treat muscle stiffness and speech impairment, which are some of the common symptoms of ALS [14]. Stem cell therapy was also proposed as a treatment for ALS to refill the amount of motor neurons that have been lost. Stem cells have the ability to renew by themselves and later get divided into the parent cell and the daughter cell [24].

The goal of this stem cell therapy was to employ stem cells and generate motor neurons to replenish the lost ones in ALS patients. However, it was proven to be clinically difficult to implement this therapy [24].

Instead, for the therapeutic treatment, riluzole was considered as the only approved treatment for ALS in the past. It is known to have an antiglutamatergic effect, inhibiting the release of glutamate at the presynaptic terminus [25]. Glutamate is an excitatory neurotransmitter, and several receptors bind to glutamate in order to instigate an action potential. Once the glutamate excitotoxicity begins, there is an increase in glutamate, uncontrolled ion channel activity, and high calcium influx. In the end, these outcomes lead to death in neurons [26]. Therefore, with the inhibition of excess glutamate production, riluzole eventually prolongs the length of survival of patients by several months [17]. However, there were some limitations reported by Miller et al., with riluzole showing some benefits on bulbar and limb function but not the muscle strength. It also had some side effects of liver problems, diarrhea, and nausea, but it was generally found to be mild in severity [26].

Another treatment option that is under study is Mastinib, which is an oral tyrosine kinase inhibitor that inhibits mast cells and neutrophils, thus preventing the damage and degeneration of neurons in the spinal cord [27]. Mora et al. carried out a randomized clinical trial regarding Mastinib as an add-on therapy to riluzole. In total, 394 patients were randomly assigned into two groups: one receiving riluzole of 100 mg/day plus placebo and a second group receiving riluzole with Mastinib 4.5 or 3.0 mg/kg/day. Primary efficacy endpoint was measuring the change in ALSFRS-R, which showed benefit for mastinib with riluzole compared to placebo with riluzole, thus proving the greater efficacy of add-on therapy of Mastinib over riluzole by itself [28]. Recently, in 2017, edaravone (Radicava) has been recognized as another treatment option for patients diagnosed with ALS as an antioxidant drug [1]. Table 2 shows the comparison between the previous approved drug, riluzole, and the recent FDA-approved drug, edaravone [29].

Other treatment options and drugs are being continued to be explored and researched. Even though there were some failures and unsuccessful trials, new therapies are improving, with new information and methods being acquired [26].

## 9. Edaravone and its Mechanism of Action

**Figure 2:**
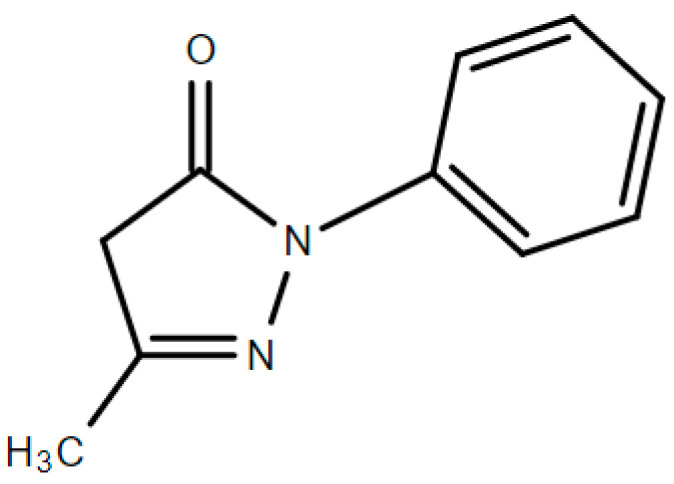
Chemical structure of edaravone.

Edaravone is a clear, colorless liquid that can be injected intravenously in a polypropylene bag with 30 mg of edaravone diluted in 100 mL of solution. The recommended dosage of edaravone is 60 mg administered via 60-min IV infusion once daily for 14 days. After this period of initial treatment, a 14-day drug-free period is required [4]. Edaravone has a chemical name of 3-methyl-1-phenyl-2-pyrazoline-5-one (Figure 2), with a code number of MCI-186. Its acid dissociation constant, pKa, is 7.0, and it is a weak acid. Edaravone also possess keto-enol tautomerism, which has a great effect on its role as a radical scavenger, having an antioxidant activity [30].

More specifically, edaravone plays a role as a free radical scavenger of peroxyl radical and peroxynitrite [31], which has a cytoprotective and neuroprotective mechanism against oxidative stress condition. Edaravone protects neurons in the brain and spinal cord by eliminating the reactive oxygen species (ROS) such as hydroxyl radical, peroxyl radical, hydrogen peroxide, peroxynitrite, and many others that quicken and exacerbate the progression of neuronal degeneration [8], thus protecting from neurologic damage that leads to the death of motor neurons [30,32].

The assembled evidence display how oxidative stress is involved in a number of diseases, including ALS, Parkinson’s disease, Alzheimer’s disease, and many other neurodegenerative diseases. Reactive oxygen species (ROS) are produced by cellular enzymes that damage components of the cells and increase inflammation. An increase of ROS is also linked to low-density lipoprotein (LDL) oxidation, thus leading to the abnormal functioning of endothelial cells in cardiovascular diseases [6].

**Figure 3:**
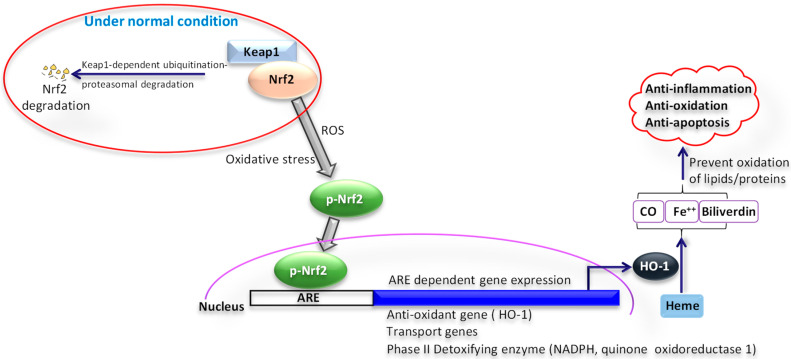
Role of nuclear factor erythroid 2-related factor 2/hemeoxygenase-1 (Nrf2/HO-1) signaling pathway in oxidative stress. Under normal condition, Nrf2 is bound to Kelch-like ECH-associated protein-1 (Keap1), gets ubiquitinated through Keap1, and undergoes proteasomal degradation. Under oxidative stress conditions, when exposed to reactive oxygen species (ROS), Keap1 inactivates and Nrf2 phosphorylation occurs. Phosphorylated Nrf2 (p-Nrf2) moves to the nucleus, where it binds to the antioxidant response element (ARE). As a result, numerous genes are activated, such as transport molecules, antioxidants, and detoxifying enzymes. This leads to an upregulation of hemeoxygenase -1 (HO-1). Upregulated HO-1 leads to a reduction of oxidative stress. HO-1 is a crucial endogenous antioxidant and creates an essential defense system. HO-1, when activated by Nrf2, can metabolize Heme into its metabolites Fe^2+^, CO, and biliverdin. These metabolites along with HO-1 by scavenging superoxide, hydroxyl-free radicals, and singlet oxygen can prevent the oxidation of proteins and lipids anions and play an important part in anti-inflammation, antioxidation, and anti-apoptosis.

As the oxidative stress and increase in ROS are involved in various pathways of human diseases, the new perception and comprehension of these linked processes will pave the way in developing new therapies and treatments for neurodegenerative diseases [6]. Evidence indicates the role of the Nrf2/HO-1 signaling pathway in protection from oxidative stress (Figure 3). The anti-inflammatory and antioxidative activities are stimulated by the nuclear transcription factor-2 erythroid related factor-2, which protects cells from oxidative stress-induced damage through antioxidant and detoxification enzymes [33]. In response to oxidative and chemical stress, the nuclear factor erythroid 2-related factor 2/antioxidant response element (Nrf2/ARE-1) signaling pathway is important in regulating antioxidants [34,35].

Several studies show that edaravone’s protective effect results from the activation of the Nrf2/HO-1 pathway, reducing cognitive damage through the activation of the Nrf2 pathway as well as acting as a defense mechanism against cell apoptosis. [34,36] Studies demonstrate that activation the NRF-2/hemeoxygenase-1 (HO-1) signaling pathway by edaravone enhances the integrity and stability of the BBB and can serve as a main target for the therapeutic treatment in in cerebral infarction [36].

Another research shows the neuroprotective effect of edaravone on the hippocampus of Kainate-induced epilepsy rat via the Nrf2/HO-1 signaling pathway. The main aim of this study was to investigate the molecular mechanism of edaravone and its anti-inflammatory effects. The result demonstrated that edaravone aided in reduction in the downregulation of mRNA and protein expression levels of Nrf2 and HO that was induced by Kainate. In addition, edaravone diminished the level of proinflammatory cytokines and NF-κB (P65) and inflammatory protein expression in the hippocampus [37].

A study was done by Yamamoto et al. 2020 to investigate the role of pH in the antioxidant activity of edaravone as a peroxyl radical scavenger. They found out that the consumption rate of edaravone increased as the pH also increased and also from the study carried out by Ohara et al., it established a proof of how edaravone’s radical scavenging activity becomes higher in high pH [38].

Another study by Kamogawa and Sueishi compared the radical scavenging rate of edaravone with other radical scavengers: hydroxyl radical, superoxide anion radical, alkoxyl radical, alkylperoxyl radical, methyl radical and singlet oxygen. Overall, edaravone was the greatest radical scavenger against hydroxyl radicals between these radical scavengers [30].

These studies of scavengers provide innovational understandings of oxidative stress and how it relates to neurodegenerative diseases as well as how we should approach in discovering new treatments [6].

## 10. Clinical Trials and the Effect of Edaravone on ALS

There have been four randomized trials that studied the safety and efficacy of edaravone. Three of those trials were double-blinded, parallel-group randomized trials conducted in Japan. The first clinical trial was named MCI186-16 and was conducted from 2006 to 2008. From then on, there have been more research and clinical trials [39]. Table 3 includes the status of clinical trials of edaravone in ALS patients.

To see the effect and the action of edaravone against ALS, Ikeda et al. carried out a study to examine the effect of edaravone on a wobbler mouse. The wobbler mouse they used was a substitute for an ALS patient as it resembled the pathology of ALS. The result showed a significant inhibition of reduction in grip strength and muscle weight, as well as showing a suppression of motor neuron degeneration in the spinal cord [38]. There were also several clinical trials that were carried out, including phase 2 and two phase 3 clinical trials regarding edaravone safety and efficacy.

The result of a phase 2 study showed a significant reduction in the score of ALSFRS-R during the treatment period of 6 months with the administration of 60 mg of edaravone compared with prior to getting treated with edaravone [30]. A phase 3 randomized double-blind study was carried out to study the safety and efficacy of edaravone at 60 mg per day administered intravenously for 2 weeks every month in early stages of ALS. This also showed a reduction in the score of Revised ALS Functional Rating Scale (ALSFRS-R) in favor of edaravone compared with the placebo group [31]. After this trial, there was an extension study also studying edaravone in ALS. In this post-hoc analyses, the population were divided into two subgroups: the efficacy-expected subpopulation (EESP) and the definite/probable EESP 2 years (dpEESP2y). Populations included in EESP need scores of ≥2 points on 12 items of ALSFRS-R with ≥80% in percentage of FVC at baseline. dpEESP2y populations need to have a definite or probable ALS with the duration of ≤2 years. In total, 181 patients were enrolled in a 12-cycle treatment. As a result, the greatest difference in ASLFRS-R score was seen in the dpEESP2y group and between edaravone and the placebo. Although there were some limitations of only involving a small number of populations in this study, post-hoc analyses all favored edaravone over the placebo, demonstrating the safety and efficacy of edaravone in ALS patients [40].

Another clinical trial was done studying the long-term efficacy of edaravone in ALS patients by Shefner et al. 2020. A phase 3 open-label study evaluated a total of 123 patients (65 taking edaravone for 48 weeks, 58 taking placebo for 24 weeks, and switching to edaravone for 24 weeks). The analyses suggested that edaravone is beneficial even after receiving placebo for 6 months, also being able to maintain the efficacy for 1 year. However, there was a limitation to this study; there were some assumptions that were made regarding the decline in ALSFRS-S score. Therefore, further research will be needed to better address the efficacy of long-term administration of edaravone [41].

Different from other previous studies, a study was done outside of Japan by Park J.M, et al. investigating the effect of edaravone therapy in Korean ALS patients. It was a small retrospective, open-label study, but it exhibited a result of modest decline in ALSFRS-R score with no known significant adverse event in patients. It also indicated that there was a significant reduction in the suppression of ALSFRS-R score irrespective of gender, onset site, and ethnicity. Finally, the study emphasized the importance of patients having an early treatment of edaravone, those with FVC of ≥60%, to see the efficacy of the drug [5].

## 11. Other Uses of Edaravone

As a free radical scavenger that provides neuroprotection, edaravone also plays a role in the treatment of stroke patients. Edaravone is also neuroprotective in Parkinson’s disease (PD). PD is another kind of neurodegenerative disease that is known to be the second most common neurodegenerative disease that is characterized by tremor, rigidity, instability in posture, and coordination [42]. It is involved with a degeneration of dopamine neurons with damage of other nerves caused by dopamine depletion within substantia nigra [6]. The exact etiology is unknown for PD; some evidence prove that it involves ROS over-generation and an extensive loss of dopamine neurons [42]. It is assumed that the mechanisms that are involved in producing and accumulating free radicals in PD include a reduction of ferritin, accumulation of iron, deficiency in glutathione, peroxidation of lipids, and dysfunction of the respiratory system [6], some of which overlaps with the pathogenesis of ALS [34], having oxidative stress and a dysfunction of mitochondrial cells being in the center of the cause of Parkinson’s disease. A study done by Yuan et al. shows that edaravone carries a neuroprotective effect on dopamine neurons, inhibiting the excess production of rotenone-induced ROS in the brain [42] and how its characteristic of being a free radical scavenger with an anti-apoptotic effect could play a role in the future for treatment of PD [43].

Another study by Nakajima et al. 2015 showed that edaravone reduces the oral mucositis score, myeloperoxidase activity, and levels of reactive substances and inflammation. Oral mucositis is an ulcerative lesion of oral mucosa, which is known to be the side effect of chemo- and radiotherapy, and it was shown that edaravone’s antioxidant effect played a protective effect in these patients [44].

Not only in PD and oral mucositis, but edaravone also shows positive effect in severe carotid territory stroke, Alzheimer’s disease, and septic myocardial dysfunction, or sepsis, referring to a systemic deleterious inflammation to an injury or an infection [45]. In one of the studies, an early administration of edaravone delayed the progression of infarction and edema in severe carotid territory stroke patients, also reducing the mortality rate during the acute phase. However, there was no significant improvement in the overall improvement in chronic function, and as a result, a combination of edaravone with antithrombosis or thrombolytic agent is recommended for these patients to improve the overall function after survival [30]. Alzheimer’s disease (AD) is also a neurodegenerative disease, sharing some common pathophysiology with PD and ALS. It is characterized by cognitive function deterioration with memory loss and behavioral impairment that could commonly be seen in these patients. Since AD is also known to be caused by the presence of excessive ROS and free radical generation that leads to cellular structural and functional degeneration, edaravone, as an antioxidant provides neutralization of free radicals, is able to inhibit the progression of neurodegenerative disorder [46].

## 12. Adverse Events and Monitoring

From U.S clinical trials and data collected from outside the U.S, one of the studies done by The Writing Group in 2017 mentions some of the common adverse events include contusion, constipation, dermatitis contact, dysphagia, eczema, and headache, which were observed in greater than 10% of the ALS patients who were treated with edaravone. However, in the study done by Park et al. 2019, these common adverse events were well-tolerated in patients with other treatments [5]. Some of the serious adverse events were hypersensitivity, dysphagia, sulfite allergic reactions, and anaphylactic symptoms. Therefore, the administration of edaravone is contraindicated in patients with a history of hypersensitivity to edaravone and those who have sulfite-related allergies [4].

Dosing adjustment for edaravone is not needed for either hepatic or renal impairment. As edaravone contains sodium bisulfite, it is important to check whether the patient is allergic prior to the administration of edaravone [26]. For the drug–drug interactions of edaravone, studies showed that therapeutic doses do not interact with other metabolites: CYP450 does not inhibit edaravone, CYP1A2, CYP2B6, or CYP3A4 also do not induce edaravone; therefore it is generally considered to be safe. With no human data available as of yet, edaravone’s risk and indication in pregnant women is unknown. The effect in lactation and infants who are breastfed is also unknown; however, animal studies indicate that edaravone leads to a decrease in growth, delay in sexual development and increase in mortality [4].

According to a drug review of edaravone by Cadth, the most common adverse event reported was respiratory failure, which led to most of the withdrawal of patients from this study. In addition, there were no outstanding harms due to the method of administration except from a catheter-site infection that was considered as a serious adverse event [39].

## 13. Discussion/Conclusions

ALS is considered a multifactorial neurodegenerative disease with several risk factors, such as environmental and genetic factors. Some of the main clinical symptoms of ALS include muscle weakness, slurred speech, muscle cramps, as well as having psychological and cognitive difficulties. With these symptoms, it is crucial to relieve the symptoms first and alleviate the patient’s quality of life. Investigations on new genes related to ALS has been ongoing, and those genes are thought to be contributing to the pathogenesis of ALS. However, despite all those years of investigation and research with more than 200 trials being done, there still is no definite cure developed for ALS. In the past, riluzole has been the main active therapy for ALS, but it had limited efficacy. More recently, only one drug was approved by the FDA for the treatment of ALS: edaravone. Other non-pharmacological therapies, such as stem cell therapy, have been proven to be well-tolerated and safe in ALS patients, but more research should be done regarding the safety and efficacy of those therapies alone or their usage in combination with other treatments.

The approval of edaravone in marketing and manufacturing was in 2015 in Japan; then, it was approved by the FDA as the treatment of ALS in 2017. Edaravone serves its neuroprotective benefit as a radical scavenger, anti-oxidant, and an anti-inflammatory against oxidative stress and ROS. In addition, with its anti-apoptotic mechanism involved with reducing Fas-associated death domain (FADD), it delays motor neuron degeneration in the brain and the spinal cord, thus prolonging the survival of patients and showing a significant improvement in ALSFRS-R score, which measures the patient’s functionality.

With ALS not having a treatment for its full cure, being able to diagnosis early and more precisely will enable earlier treatment for patients and recruit more clinical trials for future studies regarding new therapies and treatments. Although there are some limitations within the research in the field of ALS, there has been some progress shown. With extended spectrum of knowledge and research on ALS, the complexity behind neuropathology and its importance in paving the path for future treatments for patients diagnosed with ALS and other diseases that share similar pathology are beginning to be acknowledged and understood. In the future, more studies on whether the different mechanisms affecting the progression of ALS and how they relate to each other should be done, as well as the difference in the efficacy of edaravone in different stages of ALS: the early, middle, and the late stage. Furthermore, the efficacy and safety of edaravone will be strongly proved if there are more studies and clinical trials done outside of Japan, including a larger population worldwide.

As healthcare providers caring for patients, it is vital to prioritize patients’ needs and increase their quality of life. However, we should also not forget to base our decision-making on scientific evidence and research, taking the cost and efficacy of treatments and therapy being offered to patients into consideration.

## Figures and Tables

**Table 1:** Estimated prevalence of Amyotrophic Lateral Sclerosis (ALS) in United States by age, sex, and race by the National ALS Registry, United States 2015.

**Age Group (Years)**	**ALS Cases per 100,000 Population**
18–39	0.5 (Lowest prevalence)
40–49	3.6
50–59	7.4
60–69	13.5
70–79	20.2 (Highest prevalence)
**Gender**	**ALS Cases per 100,000 Population**
Male	6.4
Female	4.0
**Race**	**ALS Cases per 100,000 Population**
White	5.4
Black	2.3

**Table 2:** Simple comparison between riluzole and edaravone.

Name of a Drug	Riluzole Rilutek	Edaravone Radicava, Radicut
Molecular Formula	C8H5F3N2OS	C10H10N2O
Chemical Name	2-Amino-6-(trifluoromethoxy) benzothiazole	1-Phenyl-3-methyl-5-pyrazolone
Dose form and frequency	Oral (PO). Tablet. 50 mg every 12H. Daily.	Intravenous infusion (IV). Cycle 1: 60 mg/day for 14 days, followed by 2-week drug-free period. Cycle 2: daily dosing for 10 days, followed by 2-week drug-free period. 10 day/month.
Properties	Antiglutamatergic (inhibits glutamate release) and inactivates voltage-dependent Na channels. Exact mechanism unknown.	Free radical/(ROS) scavenger, antioxidant. Known to work against oxidative stress that cause neurodegeneration. Exact mechanism is unknown.
Side effects	Common adverse events include nausea, headache, diarrhea, liver problems.	Common side effects include contusion, confusion, constipation, eczema.

**Table 3:** Clinical trials of edaravone in ALS patients (data source ClinicalTrials.gov).

Clinical Trials Involving Edaravone Study Title	Status	Identification Code
Expanded Controlled Study of Safety and Efficacy of MCI-186 in Patients with ALS	Completed	NCT00424463
Efficacy and Safety Study of MCI-186 for Treatment of ALS	Completed	NCT00330681
Safety Extension Study of Oral Edaravone Administered in Subjects with ALS	Recruiting	NCT04577404
Efficacy and Safety Study of MCI-186 for Treatment of ALS who Met Severity Classification III	Completed	NCT00415519
Phase 3 Study of MCI-186 for Treatment of ALS	Completed	NCT01492686
Biomarkers in Different Types of ALS Patients Being Treated with Edaravone	Recruiting	NCT04097158
Treatment Effect of Edaravone in Patients with ALS	Active, not recruiting	NCT03272802
Radicava^®^ (Edaravone) Findings in Biomarkers From ALS	Recruiting	NCT04259255
Clinical Pharmacology Study of Oral Edaravone in Patients with ALS	Completed	NCT04176224
Clinical Pharmacology Study of Oral Edaravone in ALS Patients with Gastrostomy	Completed	NCT04254913
The Safety and Effectiveness of Cholinergic Receptor Block Therapy in the Treatment of ALS	Not yet recruiting	NCT04391361
Efficacy and Safety Study of Oral Edaravone Administered in Subjects with ALS	Recruiting	NCT04569084
